# Electrically Tunable Gap Surface Plasmon-based Metasurface for Visible Light

**DOI:** 10.1038/s41598-017-14583-7

**Published:** 2017-10-26

**Authors:** Jingjing Guo, Yan Tu, Lanlan Yang, Ruiwen Zhang, Lili Wang, Baoping Wang

**Affiliations:** Joint International Research Laboratory of Information Display and Visualization, School of Electronic Science and Engineering, Southeast University, Nanjing, 210096 China

## Abstract

In this paper, an electrically tunable metasuface is designed for visible regime. The device mainly consists of a V-shaped metallic metasurface, an ITO film, an electro-optic (EO) dielectric and a metal layer fabricated on a silica substrate. A continuous electrical modulation of resonant wavelength has been theoretically demonstrated in the visible range from 555 nm to 640 nm by changing the voltage applied on the EO dielectric from −20 V to 20 V. During the modulation, the steering angle also changes with the selective color. The peak cross-polarized reflectivity is higher than 48% and the bandwidth is narrower than 60 nm. The resonant wavelength shift can be explained by that the refractive index variation of the EO material induces resonance condition changes of the gap surface plasmon (GSP). The results provide a novel design solution for active plasmonic devices, especially for dynamic metadevices.

## Introduction

Metasurfaces are two-dimensional arranged metamaterials with unique properties in the spectral and spatial manipulation of electromagnetic waves. These ultra-thin planar structures offer extraordinary phase shift, amplitude modulation or polarization transition by patterning dense antenna arrays or slits, thus, its freedom on molding the light flow is larger than traditional diffraction elements which introduce multiple diffraction lobes^1–3^. Recent researches have discovered some functionalities performed by metasurfaces^4^, including frequency selective surface (FSS), polarization converter, wavefront shaping^5^ and hologram^6–10^. Most FSSs are composed of metal-dielectric nanostructures supporting surface plasmon (SP) resonances and realize a high reflectivity or refractivity, yet they also suffer from an inevitable absorption in the visible band. High-index dielectric metasurfaces, such as Si, SiN, TiO_2_ and GaP, emerge as alternative candidates to manipulate visible light due to their low energy losses in high-frequency, but they cannot be applied in an electrically-steering regime^11^.

Since the functionality of the conventional antennas is fixed after fabrication, explorations in electrically tunable metasurfaces have profound significance. Related researches have been reported utilizing liquid crystals^12–15^, graphene^16,17^, metal-doped or pure metallic dioxide assembled with indium tin oxide (ITO)^18–22^, vanadium dioxide (VO_2_)^23^, ion-attracting plasmonic crystal^24^ and so on. However, these trials mainly focus on the phase or amplitude modulation in the infrared frequency and suffer from broad spectral bandwidth and low efficiency. Explorations in the visible light is handful so far and cannot realize a flexible steering on colors.

In this paper, we propose a novel electrically tunable metasurface configuration. The reflective color can be adjusted continuously in the visible regime by electric modulation. A dielectric material with large EO coefficient is adopted in the GSP-based metasurface in the developed device to implement a resonance condition shift due to its electrically tunable characteristics. The cross-polarized resonance reflective wavelength can be tuned theoretically from 555 nm to 640 nm by changing the voltage applied to the EO material from −20 V to 20 V. We hope this design could be applied in dynamic meta-display and hologram.

## Results

A GSP-based gradient metasurface is a metal-dielectric-metal configuration with metallic nanobrick arrays on its top layer. The bottom metal layer behaves as a mirror^25,26^. The resonance of electromagnetic waves has been verified and explained by the standing-waves of GSPs, which follows the typical Fabry-Perot resonator formula

1 $$L{k}_{0}{n}_{gsp}+\varphi =m\pi .$$

here *L* is the width of the nanobrick, *k*_0_ is the vacuum wave number, *n*_*gsp*_ is the effective refractive index of the GSP, *m* is an integer defining the mode order, and *ϕ* is an additional phase shift. Herein, *n*_*gsp*_ is depended on the refractive index of metal and dielectric, and the width of the dielectric gap^27^. In this paper, we use metallic antenna arrays, an EO dielectric and a metal layer to form a GSP resonator. The refractive index of the EO material, such as 4-dimethyl-amino-Nmethyl-4-stilbazolium tosylate (DAST)^28,29^, can be changed via different voltages applied to the EO material. Therefore, the resonance frequency of the device can be electrically manipulated.

**Figure 1:**
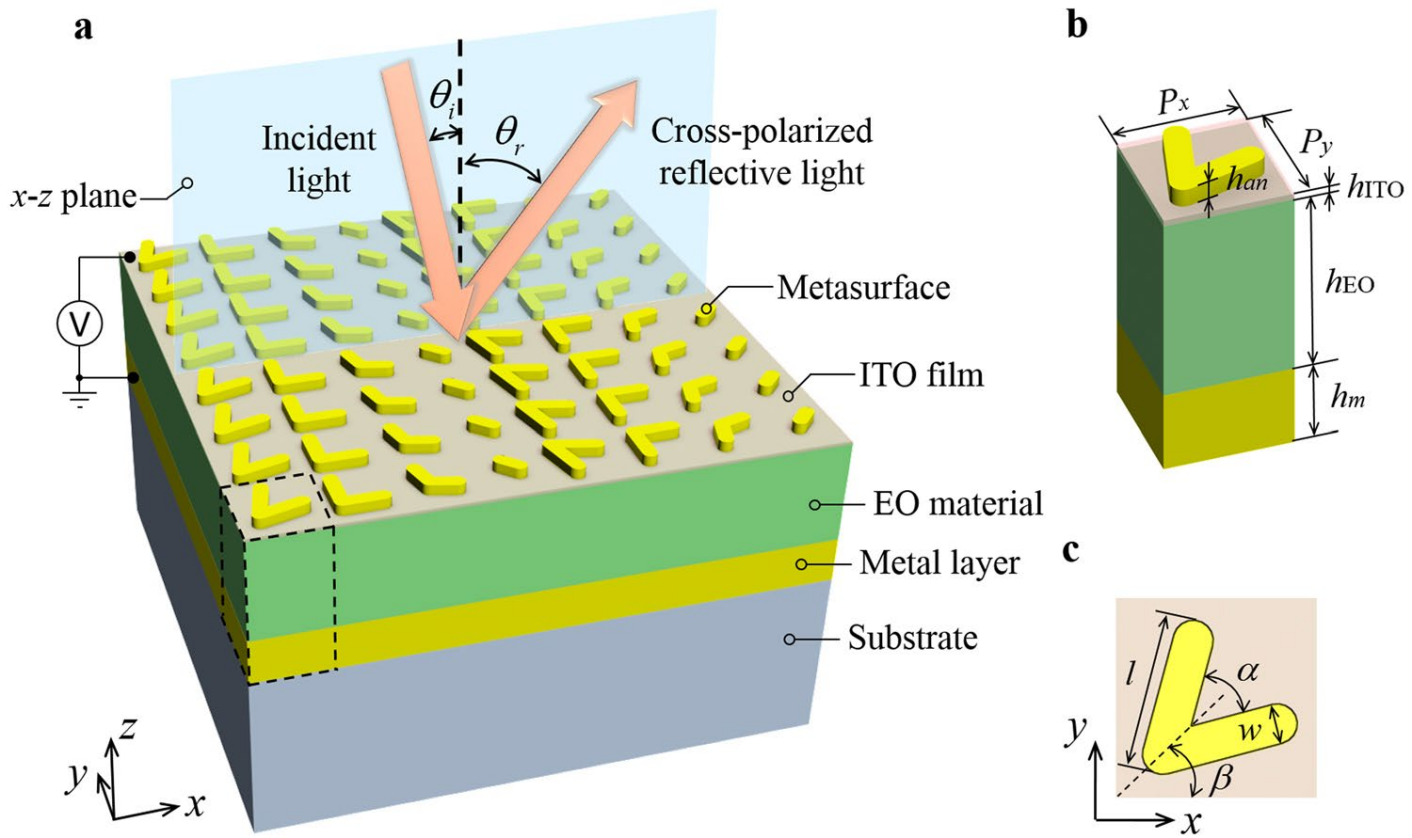
Schematic of electrically-tuned metasurface. (**a**) A metallic V-shaped metasurface, an EO material layer and a metal layer form a GSP resonance cavity. External voltage is applied on the ultrathin ITO film adjacent to the bottom interface of the metasurface while the metal layer acts as a grounding electrode. A period of metasurface comprises eight antenna unit cells, being arranged parallel to *x*-axis. (**b**) A unit cell of the GSP cavity in the dashed frame of Fig. 1a. The period in *x-* and *y-*direction is *P*_*x*_ and *P*_*y*_, respectively. The antenna, the ITO film, the EO material and the metal layer has a corresponding height of *h*_*an*_, *h*_ITO_, *h*_EO_ and *h*_*m*_. (**c**) A V-shaped antenna has two *l*-long, *w*-wide arms splitting with an angle *α*. The angle between the symmetric axis and *x*-axis is *β*.

A scheme of our novel electrically tunable metasurface device is shown in Fig. 1. It is composed of a V-shaped metasurface, an ITO film, an EO material layer and a metal layer deposited on a silicon substrate. Herein the transparent conducting ITO film is attached below the metasurface, acting as an electrode. Voltage applied on the EO dielectric induces a refractive index shift. This shift leads to a change of both *n*_*gsp*_ and *ϕ* (in Eq. 1), and finally modulates the resonance wavelength. Here the metal layer is connected to the ground. Generally, geometry-varied metasurface arouses anomalous reflection and refraction for linear polarized light while orientation-varied one for circular polarized light. And different antenna profiles function for different intentions. We choose geometry-varied V-shaped antennas to obtain a narrow bandwidth FSS. When the *x*-polarized waves illuminate onto the metasuface at normal incidence, an abrupt phase shift is introduced along the *x*-axis by the nano-antennas, and the *y*-polarized reflective light axis is oblique in the *x*-*z* plane. The anomalous reflective angle *θ*_*r*_ is decided by the generalized Snell’s law of reflection^1^

2 $${\theta }_{r}=\arcsin [\sin ({\theta }_{i})+\frac{\lambda }{{n}_{i}{\rm{\Gamma }}}]$$

where *θ*_*i*_ is the incident angle (*θ*_*i*_ = 0 in this paper); *λ* is the free-space wavelength; *n*_*i*_ is the refractive index of the incident region (*n*_*i*_ = 1 in our simulation); Γ is the period of the antenna array. There are eight geometry-varied V-shaped antenna unit cells with a period of 200 nm both in *x*- and *y*-direction (*P*_*x*_ and *P*_*y*_ in Fig. 1b), and all of them are arranged in a line parallel to the *x*-axis. So the period of the antenna array Γ = 8*P*_*x*_. The compact arrangement of the unit cells results from the demand of visible working frequency. The corresponding height of the antennas, the ITO film, the EO material layer and the metal layer is *h*_*an*_, *h*_ITO_, *h*_EO_ and *h*_*m*_, respectively. The metal layer *h*_*m*_ equals to 130 nm in our simulations, which is thick enough to totally reflect GSPs^7^. For an individual antenna, as shown in Fig. 1c, two rectangle arms split at an angle *α* forming a V shape. The arm has a length of *l* and a width of *w*. The angle between the symmetric axis and *x*-axis is *β*.

**Figure 2:**
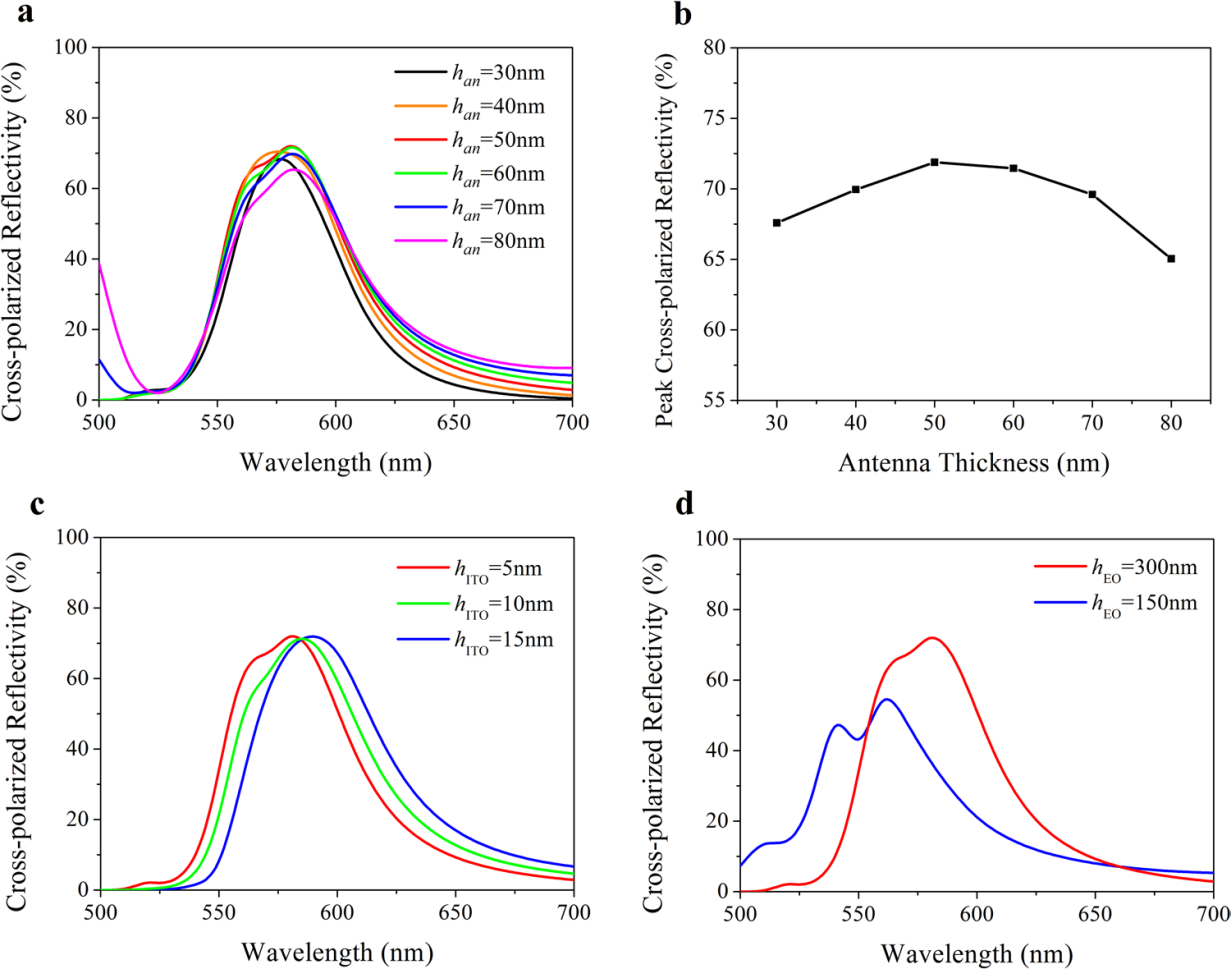
Optical properties of a unit cell under no voltage bias. (**a**),(**c**) and (**d**) Cross-polarized reflective spectrum with the different thickness of the antenna, the ITO film and the EO material. (**b**) Peak cross-polarized reflectivity as a function of antenna thickness. All the discussions are based on the control variable method with the reference parameters of *h*_*an*_ = 50 nm, *h*_ITO_ = 5 nm and *h*_EO_ = 300 nm.

First, we optimize the characteristics of a V-shaped unit cell without applied voltage on the EO material. The calculated spectral behavior color selectivity of a unit cell is plotted in Fig. 2 using control variable method when *l* = 147 nm, *w* = 40 nm, *α* = 60° and *β* = 45°. Under no voltage condition, as shown in Fig. 2a, the resonance condition is adjusted by different thickness of the antenna. Secondary resonant peaks appear when the antenna thickens up to 70 nm, which is unexpected for color selectivity. Also, in Fig. 2b, a too thick or too thin antenna weakens the peak reflectivity. This value reaches a maximal of 72% when the antenna thickness is 50 nm. So we choose 50 nm for further analysis. In terms of *h*_ITO_ and *h*_EO_, they mainly influence the additional phase (*ϕ* in Eq. 1). In Fig. 2c, the resonance peak shifts 5 nm to the longer wavelength region as *h*_ITO_ thickening per 5 nm while the peak reflectivity remains unchanged. The narrowest bandwidth is 55 nm with *h*_ITO_ of 5 nm. So a 5nm-thick ITO film is optimal. Finally, as shown in Fig. 2d, a too thin EO material may decrease the peak cross-polarized reflectivity. This is corresponding to the GSP theory that a more compact cavity arouses a stronger confining GSP mode, leading to more absorption in the cavity and less energy coupled out^26^. Nevertheless, the thickness of EO material is also limited to the maximal electric field intensity *E*. Taking DAST into account, its refractive index variation is proportional to *E*. The GSP cavity forms almost a uniform electric field, and *E* inversely proportional to distance *h*_EO_. Thus a thicker EO dielectric requires higher voltage to realize the same resonance wavelength shift. Therefore, a 300 nm-thick EO material is used in our design to ensure a relative low voltage modulation and a reasonable peak cross-polarized reflectivity.

**Figure 3:**
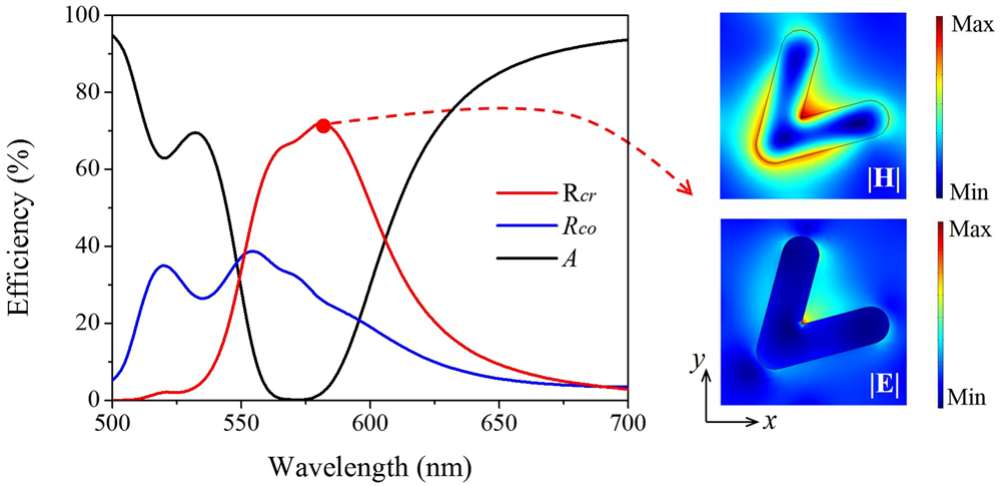
Spectral distribution when *h*_*an*_ = 50 nm, *h*_ITO_ = 5 nm and *h*_EO_ = 300 nm. *R*_*co*_, *R*_*cr*_ and *A* represents co-polarized reflectivity, cross-polarized reflectivity and absorption efficiency, respectively. Insets are the enhancement of the magnetic and electric field magnitude in the middle of the nano-antenna.

Accordingly the structure parameters are now optimized to *h*_*an*_ = 50 nm, *h*_ITO_ = 5 nm and *h*_EO_ = 300 nm. At this condition, Fig. 3 signifies that free-space propagating waves are coupled into SPs in a spectrum range from 545 nm to 610 nm. Then the SPs are partially absorbed in the GSP resonator and other part of them are coupled out and converted into free propagating waves with cross polarization. Herein, the main cross-polarized reflective peak wavelength is 580 nm with a value of 72% and a bandwidth of 55 nm. The magnetic and electric field norm distribution at 580 nm is shown in the insets of Fig. 3. A reinforced magnetic field can be observed at the corners of the antenna, dominated by the SPs. Additionally, the wavelength of SPs confined to the resonator is shorter than the out-coupling radiations’. This is a phase mismatching condition for the chosen unit cell period, yet does not influence the optical characteristics of the anomalous reflection waves.

**Figure 4:**
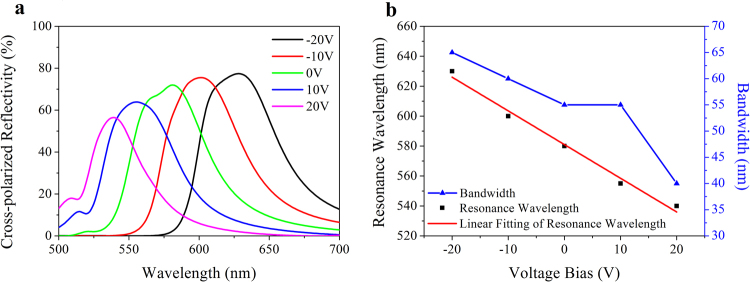
Voltage-induced resonance wavelength shift. (**a**) Spectral distribution under different voltage bias. (**b**) Resonance wavelength and bandwidth as a function of voltage bias.

Then the influence of voltage on the optical characteristics is investigated. As shown in Fig. 4a, a continuous color selectivity is realized by electrical modulation. The peak reflectivity gradually decreases from 77% to 56% when the voltage bias is adjusted from −20 V to 20 V, due to the absorption enhancement aroused by the index increment of DAST. As shown in Fig. 4b, the resonance wavelength *λ*_*r*_ and the simulated voltage *U* present a distinct linear relationship. The linear regression equation is fitted as *λ*_*r*_ = −2.25 (nm/V)∙*U* + 581(nm) with the R-square of 0.985, which means the resonance peak shifts about 22.5 nm per 10 V. The bandwidth of the reflective spectrum decreases from 65 nm to 60 nm when the negative voltage bias decreases from −20 V to −10 V. This value remains 55 nm under no voltage bias and 10 V, and finally decline to 40 nm under 20 V.

**Figure 5:**
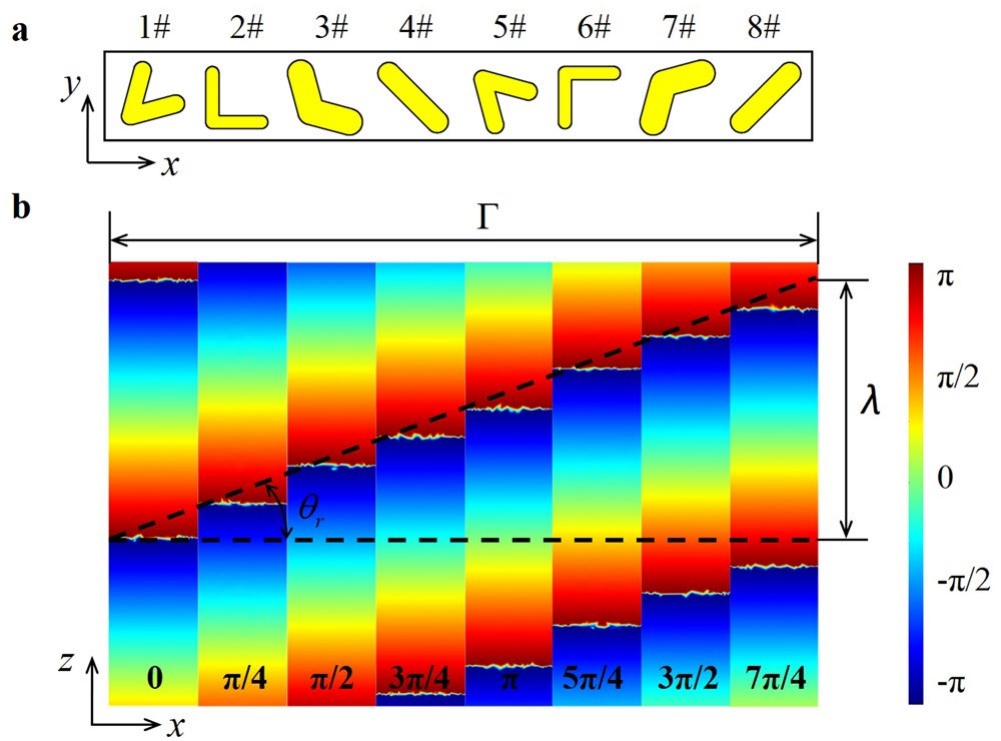
Designed antenna arrays. (**a**) Geometry of our antenna array. (**b**) Calculated scattered electric field phase of the individual antennas in the *y* direction for *x*-polarized incident plane wave.

To interpret the phase modulation of our antenna more clearly, a phase gradient metasurface is designed. Based on the analysis of the unit cell, the antennas are engineered to realize 2π full control of the wavefront and a phase gradient (2π/Γ) along the interface of the metasurface. The schematic antenna array is depicted in Fig. 5a. In an array period, the first four antennas (label from 1# to 4#) have been optimized to *β* = 45°, *α* = 60°, 90°, 120°, 60°, *l* = 147 nm, 141 nm, 150 nm, 205 nm and *w* = 40 nm, 30 nm, 60 nm, 50 nm. The latter four antennas (label from 5# to 8#) have *β* = −45° and other parameters are consistent with the first four unit cells. This is because that when the symmetric axis rotates 90°, its corresponding reflective phase adds π. The scattered electric field above the antennas is illustrated in Fig. 5b. The un-shown background field is an *x*-polarized plane wave with a wavelength of 580 nm incident from the top air. An oblique plane wave for cross-polarization is generated on account of a superposition of the reflection beams of these individual antennas, wherein the refractive angle satisfies the generalized Snell’s law (Eq. 2).

**Figure 6:**
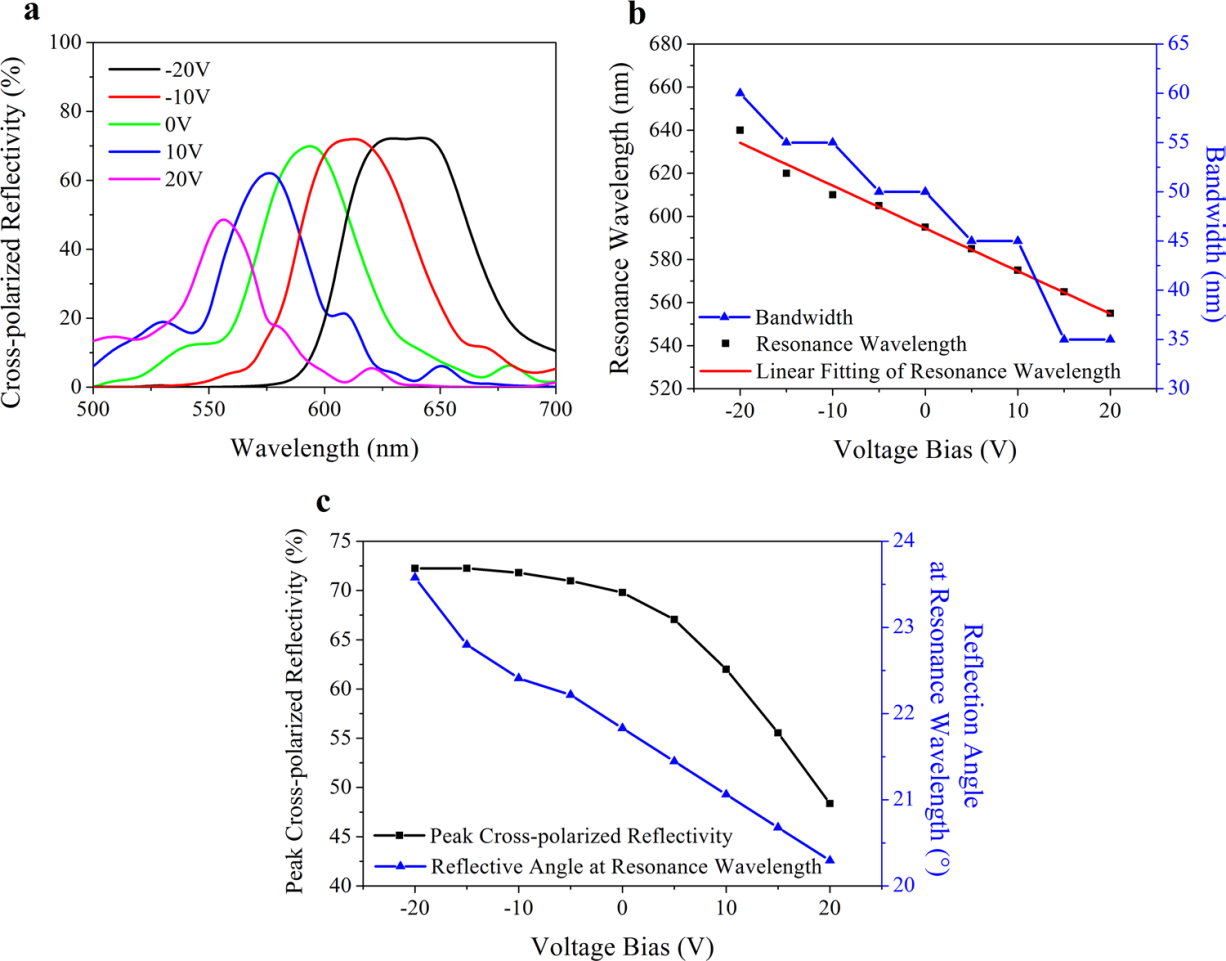
Voltage-tuned color selectivity of the designed metasurface. (**a**) Spectral behavior for different voltage bias. (**b**) Resonance wavelength and bandwidth as a function of applied voltage bias. (**c**) Steering angle changes with voltage bias.

The functionality of electrical modulation is plotted in Fig. 6. A significant wavelength shift induced by voltage bias is shown in Fig. 6a. Similar to one unit cell analysis, the resonance wavelength *λ*_*r*_ presents a significant linear relationship with the simulated voltage *U*, as plotted in Fig. 6b. The linear regression equation is fitted as *λ*_*r*_ = −1.98 (nm/V)∙*U* + 594.4 (nm) with the R-square of 0.986, which means the resonance peak approximately shifts 19.8 nm per 10 V, less than that of the unit cell. In contrast with one antenna, the bandwidth drops from 60 nm to 35 nm under voltage bias range from −20 V to 20 V. As shown in Fig. 6c, the peak cross-polarized reflectivity decreases with the blue shift of the resonance wavelength. The maximal reflectivity 72% occurs in a wavelength of 640 nm under −20 V while the minimum is 48% for 555 nm under 20 V. When the operating wavelength is tuned from blue to red, the steering angle changes from 23.6° to 20.3° as well. The changes of the reflection angle satisfy the generalized Snell’s law (Eq. 2). These results demonstrate that our design realizes an effective electric modulation on the anomalous reflective wavelength of the gradient metasurface. This design can be possibly used in color display and holographic imaging.

## Method

All modelling results are performed using finite-element-method. In the simulations, only one antenna period with 1μm-thick air layer above is considered with periodic boundary conditions on the side walls in the *x*- and *y*- directions. Perfect matched layers are applied on the *z* direction to absorb all the scattering waves. The incident wave is assumed to be a plane wave propagating normal to the metasurface with TM polarization. The refractive index of silver is interpolated from experimental values by Johnson^30^. The permittivity *ε* of the ITO film is determined by the Drude model, which is given by

3 $$\varepsilon ={\varepsilon }_{\infty }-\frac{{{\omega }_{p}}^{2}}{{\omega }^{2}+i{\rm{\Gamma }}\omega }$$

where *ε*_∞_ is permittivity at infinite frequency, *ω* is angular frequency, *ω*_*p*_ is plasma frequency, and Γ is relaxation frequency. The corresponding value of *ε*_∞_, *ω*_*p*_ and Γ is 4.55, 2.0968 × 10^15^ rad/s and 724.6 THz^31^. The refractive index of the ITO film can be retrieved from the square root of *ε*. EO material is an organic crystal DAST with a linear refractive index (*n*_0_ = 2.2) and a large EO coefficient (*dn*/*dE* = 3.41 nm/V). The relation between the voltage and the refractive index of EO material is *n* = *n*_0_ +  (*dn*/*dE*) ∙ (*U*/*h*_EO_), where *U* is the applied voltage^28,29,32^. In the static electric simulation, the surfaces of the antennas and the ITO film is assumed as equipotential surfaces, acting as electrodes. And the metal layer is connected to the ground. Here the relative static permittivity of ITO, DAST and silver is 9.3, 5.2 and 1, respectively. The reflectivity is calculated by the intensity of the reflective light for a certain polarization normalized to the incident light, of which the light intensity is obtained by integrating the square of the electric field intensity along a parallel plane in the air domain 1um away from the metasurface. In detail, an all-air-model is firstly established to record the incident field intensity. Next, the simulation model with all the designed materials and structure is built. Then we proceed to the static electric simulation to get the refractive index of the EO material, and finally turn to the scatter field calculation.

### Data availability

The datasets generated and analyzed during the current study are available from the corresponding author on reasonable request.
